# Assessment of fever screening at airports in detecting domestic passengers infected with SARS-CoV-2, 2020–2022, Okinawa prefecture, Japan

**DOI:** 10.1186/s12879-024-09427-5

**Published:** 2024-05-30

**Authors:** Yoshihiro Takayama, Yining S. Xu, Yusuke Shimakawa, Gerardo Chowell, Masahiro Kozuka, Ryosuke Omori, Ryota Matsuyama, Taro Yamamoto, Kenji Mizumoto

**Affiliations:** 1 Okinawa Prefecture Commission for Epidemiological and Statistical Analysis, Naha-shi, Okinawa Japan; 2 Okinawa Chubu Hospital, 281, Miyazato, Uruma, Okinawa 904-2293 Japan; 3 Department of International Health and Medical Anthropology, Institute of Tropical Medicine, Nagasaki University, 1-12-4 Sakamoto, Nagasaki, 852-8523 Japan; 4 Graduate School of Advanced Integrated Studies in Human Survivability, Kyoto University, 1, Yoshida-Nakaadachi-Cho, Sakyo-Ku, Kyoto, 606-8306 Japan; 5 Unité d’Épidémiologie des Maladies Émergentes, Institut Pasteur, Université Paris Cité, 25-28 rue du Docteur Roux, Paris, 75724 France; 6 International Research Center for Medical Sciences, Kumamoto University, 2-2-1, Honjo, Chuo-ku, Kumamoto, 860-0811 Japan; 7 School of Public Health, Georgia State University, 33 Gilmer Street SE, Atlanta, GA 30303 USA; 8 Division of Bioinformatics, International Institute for Zoonosis Control, Hokkaido University, North 20, West 10 Kita-ku, Sapporo, Hokkaido 001-0020 Japan; 9 Rakuno Gakuen University, 582, Bunkyodai Midorimachi, Ebetsu, Hokkaido 069-0836 Japan; 10 Hakubi Center for Advanced Research, Kyoto University, Yoshidahonmachi, Sakyo-Ku, Kyoto, 606-8501 Japan; 11 Pasteur International Unit at Kumamoto University / National Center for Global Health and Medicine, Tokyo, Japan

**Keywords:** Fever screening, COVID-19, Airport, Prevention

## Abstract

**Background:**

While airport screening measures for COVID-19 infected passengers at international airports worldwide have been greatly relaxed, observational studies evaluating fever screening alone at airports remain scarce. The purpose of this study is to retrospectively assess the effectiveness of fever screening at airports in preventing the influx of COVID-19 infected persons.

**Methods:**

We conducted a retrospective epidemiological analysis of fever screening implemented at 9 airports in Okinawa Prefecture from May 2020 to March 2022. The number of passengers covered during the same period was 9,003,616 arriving at 9 airports in Okinawa Prefecture and 5,712,983 departing passengers at Naha Airport. The capture rate was defined as the proportion of reported COVID-19 cases who would have passed through airport screening to the number of suspected cases through fever screening at the airport, and this calculation used passengers arriving at Naha Airport and surveillance data collected by Okinawa Prefecture between May 2020 and March 2021.

**Results:**

From May 2020 to March 2021, 4.09 million people were reported to pass through airports in Okinawa. During the same period, at least 122 people with COVID-19 infection arrived at the airports in Okinawa, but only a 10 suspected cases were detected; therefore, the capture rate is estimated to be up to 8.2% (95% CI: 4.00-14.56%). Our result of a fever screening rate is 0.0002% (95%CI: 0.0003–0.0006%) (10 suspected cases /2,971,198 arriving passengers). The refusal rate of passengers detected by thermography who did not respond to temperature measurements was 0.70% (95% CI: 0.19–1.78%) (4 passengers/572 passengers).

**Conclusions:**

This study revealed that airport screening based on thermography alone missed over 90% of COVID-19 infected cases, indicating that thermography screening may be ineffective as a border control measure. The fact that only 10 febrile cases were detected after screening approximately 3 million passengers suggests the need to introduce measures targeting asymptomatic infections, especially with long incubation periods. Therefore, other countermeasures, e.g. preboarding RT-PCR testing, are highly recommended during an epidemic satisfying World Health Organization (WHO) Public Health Emergency of International Concern (PHEIC) criteria with pathogen characteristics similar or exceeding SARS-CoV-2, especially when traveling to rural cities with limited medical resources.

**Supplementary Information:**

The online version contains supplementary material available at 10.1186/s12879-024-09427-5.

## Background

Infectious diseases with human-to-human transmission potential, such as respiratory diseases (e.g., influenza, COVID-19), spread to other areas through human mobility [1]. For example, soon after the world faced the imminent threat of COVID-19, strict travel restrictions were initiated worldwide. Various types of stringent border controls were implemented to prevent the influx of infected persons and the subsequent spread of the disease within the country/region.

In terms of human mobility speed, which is directly linked to the spread of infectious diseases, the impact of airplanes stands out from other modes of transportation. Therefore, in addition to frequently used fever screening, other strong border control measures were implemented at international airports, which are the air gateways to many countries. Those measures included restricting entry from epidemic areas by visa, requiring a valid vaccination certificate, pre-departure test, on-arrival test, and waiting in a designated facility for a certain period of time after entry into the country [2].

As symbolized by the declaration of an end to the coronavirus crisis as a “public health emergency of international concern” by the World Health Organization on May 5, 2023, as the public health threat of COVID-19 decreases, control measures, including border control measures at airports, have eased [3]. After April 29, 2023, only genomic surveillance of infectious diseases focused on arriving passengers in Japan was conducted, targeting symptomatic patients willing to cooperate in the survey [2].

Unlike the 2009 H1N1 Pandemic, various border control measures such as RT-PCR testing before and after entry and requiring a vaccination certificate were imposed against COVID-19. Therefore, observational studies documenting the effectiveness of fever screening at airports against COVID-19 alone have been limited [4, 5], with a modeling study estimating the percentage of missed infections as high as 46% [6], while fever screening at airports was shown to be largely ineffective during the 2009 A/H1N1 influenza pandemic [7–9]. Several conditions must be met to evaluate the capture rate of infected persons by thermography alone through airport screening. Specifically, the geographic location of interest should not border other countries/regions without considering the influence of overland routes such as cars and rail, which are usually the main routes. Other requirements include the need for a large-scale laboratory system to test possible infected individuals and record all patient information, such as residential area and travel history, throughout an epidemic that has tremendously burdened the health care system. Just as Japan is not connected to other countries by land and the main route of entry is by air, Okinawa Prefecture is not connected to other prefectures by land, and the main route of entry is by air. Unlike the United Kingdom and Hokkaido Prefecture in Japan, Okinawa Prefecture has no routes of entry through undersea tunnels to neighboring countries or regions. Moreover, large RT-PCR centers, including free-of-charge testing, were established for monitoring purposes inside the prefecture [10]. In addition, since the beginning of the epidemic, Okinawa Prefecture has collected information on COVID-19 patients, including the residences and travel histories of those confirmed with SARS-CoV-2.

The purpose of this study is to retrospectively assess the effectiveness of fever screening at airports in preventing the influx of COVID-19-infected persons as airport screening measures for COVID-19 infected passengers around the world have been greatly relaxed.

## Methods

### Study setting

At the southwestern tip of the Japanese archipelago, Okinawa Prefecture consists of more than 160 islands of various sizes in a vast sea area. The number of airports in Japan is 82, of which 13 (15.9%) are in Okinawa Prefecture [11]. Geographically far from other prefectures, travel from other prefectures to Okinawa Prefecture is by air and sea, with approximately 22 million air passengers and over 2.3 million sea passengers reported yearly as of 2019, before the COVID-19 pandemic [12–14]. According to the most recent 2018 report with available data, approximately 8.67 million (88%) of the tourists entering Okinawa Prefecture were by air and 1.17 million (12%) by sea, with foreign visitors using cruise ships accounting for 1.12 million (96%) of the sea visitors [15, 16]. Following events of COVID-19 spread on cruise ships in early 2020 [17–20], cruise ships were suspended during the pandemic and reopened until March 2023.

Okinawa’s population comprises approximately 1.45 million people, of whom 1.32 million (91%) are on the main islands and 130,000 (9%) are on the outlying islands. Tourism is one of the main industries, accounting for 20% of the prefecture’s Gross Domestic Product (GDP). As of 2019, the number of tourists was 10.2 million per year, about the same number as Hawaii in the United States, which is also famous for tourism [21, 22].

**Fig. 1:**
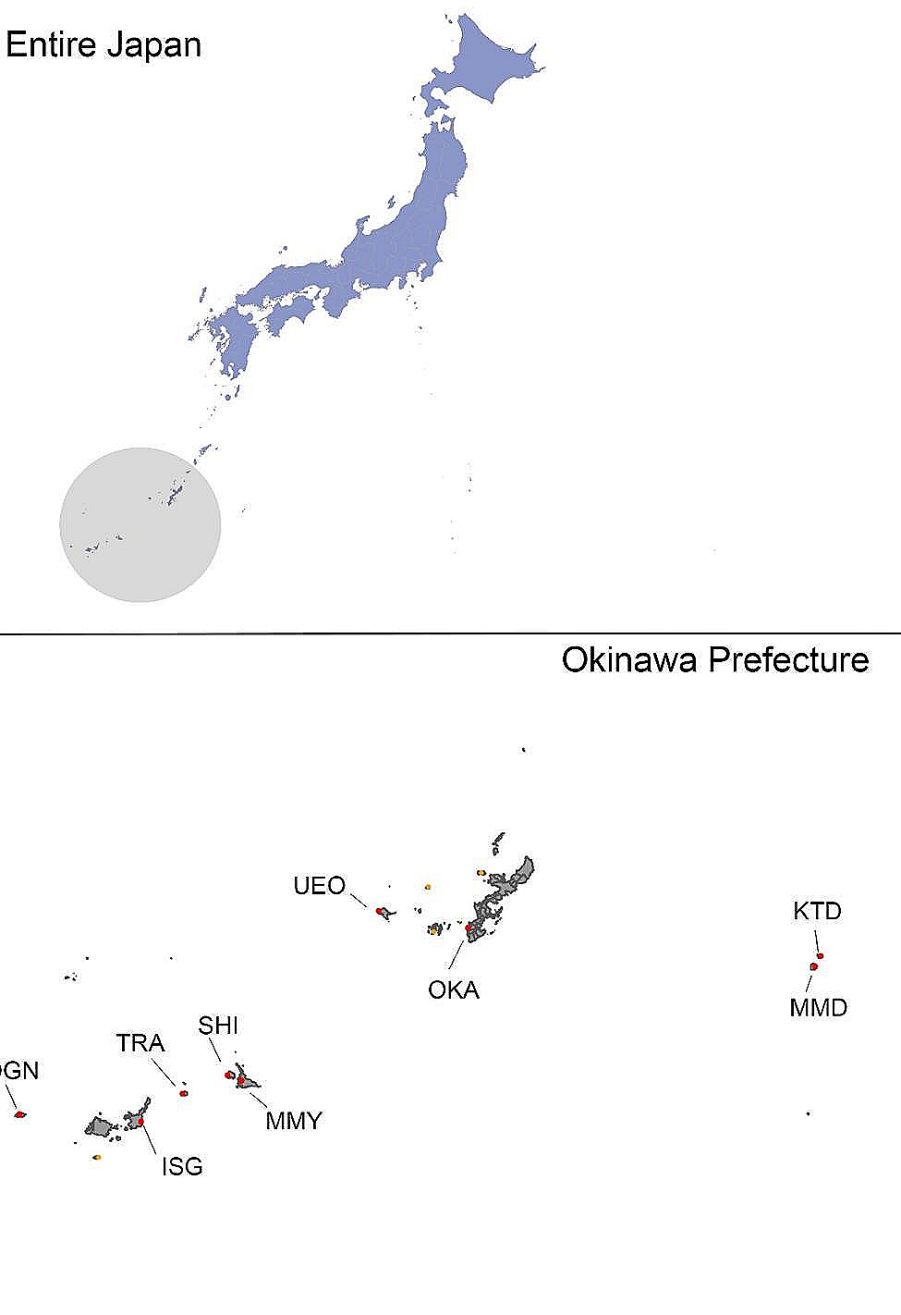
Location of Okinawa Prefecture and nine airports conducted airport screening The nine airports participating in this study are illustrated by red dots: OKA: Naha Airport, MMY: Miyako Airport, ISG: Ishigaki Airport, UEO: Kumejima Airport, TRA: Tarama Airport, OGN: Yonaguni Airport, KTD: Kitadaito Airport, MMD: Minamidaito, SHI: Shimoji Airport The remaining airports are illustrated by pink dots

In Okinawa Prefecture, thermographic airport screening at airports was conducted from May 2020, when the COVID-19 epidemic spread, until September 2022. Airport screening for arriving passengers was conducted at 9 of the 13 airports in the prefecture (Fig. 1). At Naha Airport, screening was also conducted for departing passengers.

The nine airports participating in this study are illustrated by red dots: OKA: Naha Airport, MMY: Miyako Airport, ISG: Ishigaki Airport, UEO: Kumejima Airport, TRA: Tarama Airport, OGN: Yonaguni Airport, KTD: Kitadaito Airport, MMD: Minamidaito, SHI: Shimoji Airport. The remaining airports are illustrated by pink dots.

**Fig. 2:**
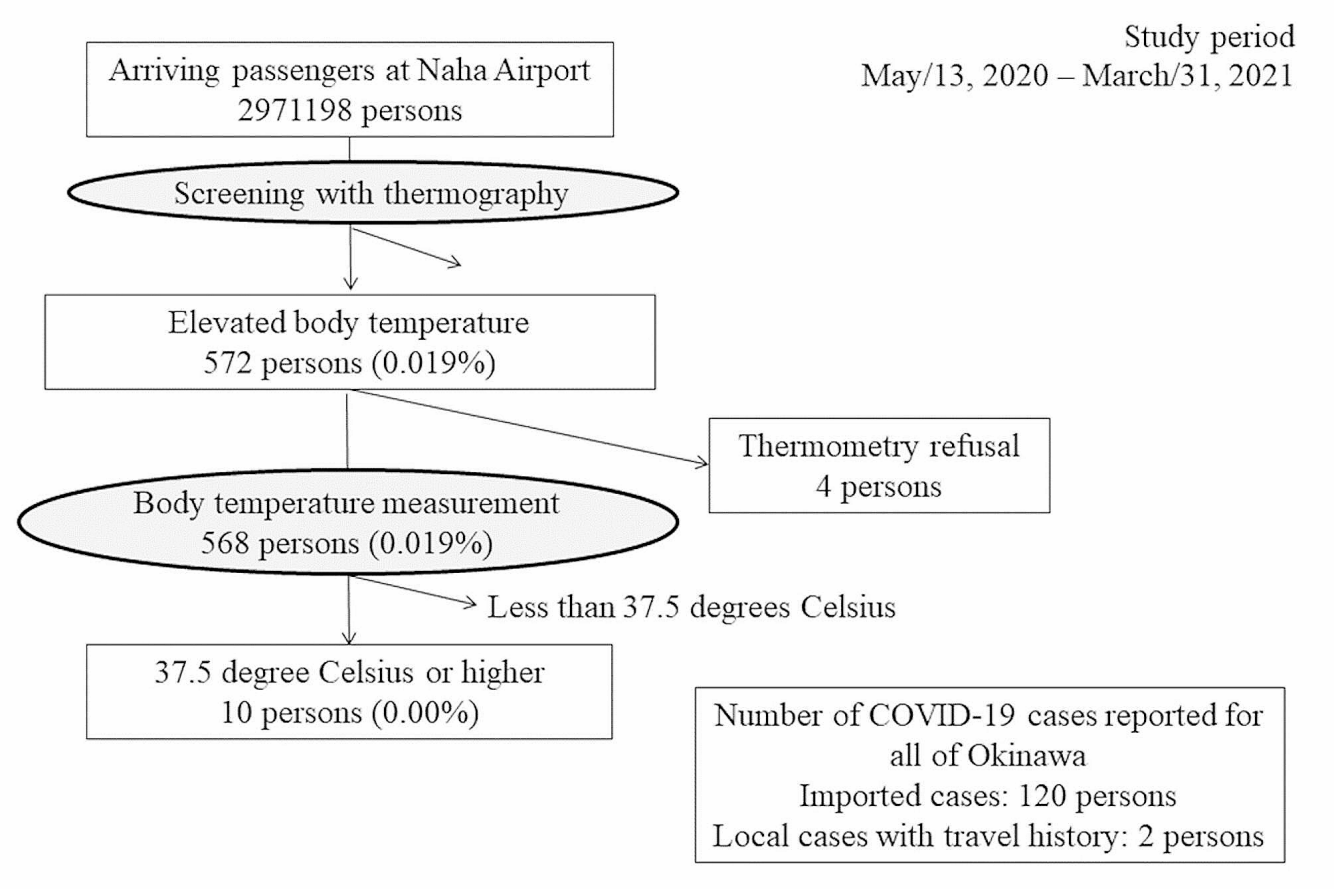
Flow diagram of the airport screening for arriving passengers at Naha Airport All the arriving passengers were screened by thermography on arrival. Arriving passengers undergo a thermographic body temperature check as they exit the baggage claim area and enter the arrival gate. For follow-up, persons whose body temperature is detected by thermography are requested to take their temperature using a thermometer by the examiner. A thermometer will be used to determine if febrile or not. Febrile passengers are requested to provide individual information and to undergo diagnostic testing at the RT-PCR testing center adjacent to the airport

A flowchart of airport screening at Naha Airport is presented in Fig. 2. All arriving passengers underwent systematic screening using thermography upon arrival. A thermographic body temperature check was performed when passengers entered the baggage claim area toward the arrival gate. Persons identified by thermography as potentially having elevated body temperature were requested to confirm their temperature using a non-contact thermometer directed to the forehead or wrists. Although there was no standard protocol related to the measurement of body temperature, the contractor contracted by the prefecture verified that there was no difference between the body temperature measured by the reference non-contact thermometer and the body temperature measured by thermography. Since the temperature at which the thermography detects a fever can be affected by outside temperatures and other factors, the temperature setting was changed on August 15, 2020, from the original 37.0 °C to 36.5 °C, to avoid missing febrile passengers. In this analysis, the definition of fever was 37.5 °C or higher as defined by Japan’s Infectious Diseases Control Law. Febrile passengers were then asked to provide individual information and to undergo diagnostic testing at the RT-PCR testing center adjacent to the airport.

All the arriving passengers were screened by thermography on arrival. Arriving passengers undergo a thermographic body temperature check as they exit the baggage claim area and enter the arrival gate. For follow-up, persons whose body temperature is detected by thermography are requested to take their temperature using a thermometer by the examiner. On August 15, 2020, the setting for thermography to detect elevated body temperature was changed from the original 37.0 °C to 36.5 °C. A thermometer will be used to determine if it is febrile or not. Febrile passengers are requested to provide individual information and to undergo diagnostic testing at the RT-PCR testing center adjacent to the airport.

### Data sources

Time series data on the number of passengers and thermographically detected passengers stratified by airport were provided by Okinawa Prefecture. Data on the number of febrile passengers arriving at Naha Airport by thermometer measurement were also provided for the period through March 31, 2021. An explanation for the refusal to take body temperature was documented for refusing passengers. RT-PCR test results have not been systematically obtained for febrile individuals because no law mandates testing.

Definitions of local cases with travel history and imported cases are those whose place of residence is Okinawa Prefecture, those who traveled outside Okinawa Prefecture just before the diagnosis, and those whose place of residence is outside Okinawa, respectively. Data on imported cases and local cases with a travel history were retrieved from COVID-19 case data collected by Okinawa Prefecture through surveillance, including date of confirmed diagnosis, place of residence, and travel history. During the study period between May 13, 2020 and March 31, 2021, 122 COVID-19 confirmed cases were reported (120 imported cases and 2 local cases with travel history).

The maximum temperatures at the airport were obtained from the Japan Meteorological Agency, and the temperature measurement points were selected from the island where each airport is located. If there are multiple measurement points on the island, the one geographically closest to the airport was selected [23].

### Statistical analysis

The thermographic detection rate was defined as the proportion of passengers suspected of having an elevated temperature based on thermographic screening. The capture rate was defined as the proportion of reported COVID-19 cases who would have passed through airport screening (a total of local cases with travel history and imported cases) to the number of suspected cases (febrile cases) through fever screening at airports. Confidence intervals were obtained using the exact binomial test. All statistical analyses were conducted using R version 4.2.3 (R Foundation for Statistical Computing, Vienna, Austria).

## Results

The results of airport screening for arriving passengers at Naha Airport from May 13, 2020, to March 31, 2021, are summarized in Fig. 2. Of the 2,971,198 arrivals, 572 individuals were suspected of elevated temperature based on thermographic screening (thermographic detection rate: 0.02%, 95%CI: 0.02–0.02%). Out of the 572 passengers detected by thermography, 4 passengers did not comply with the request for temperature measurement using a thermometer, resulting in a refusal rate of 0.70% (95%CI: 0.19–1.78%) (4 passengers/572 passengers). Among the 568 passengers whose temperature was measured using a thermometer, ten had a body temperature of 37.5 °C or higher (1.76%, 95%CI: 0.85–3.21%). Considering that there were a total of 122 COVID-19 confirmed cases was reported (120 imported cases and 2 local cases with travel history) during the study period, the estimated capture rate was 8.2% (95%CI: 4.00-14.56%) (10 suspected cases /122 confirmed cases).

**Fig. 3:**
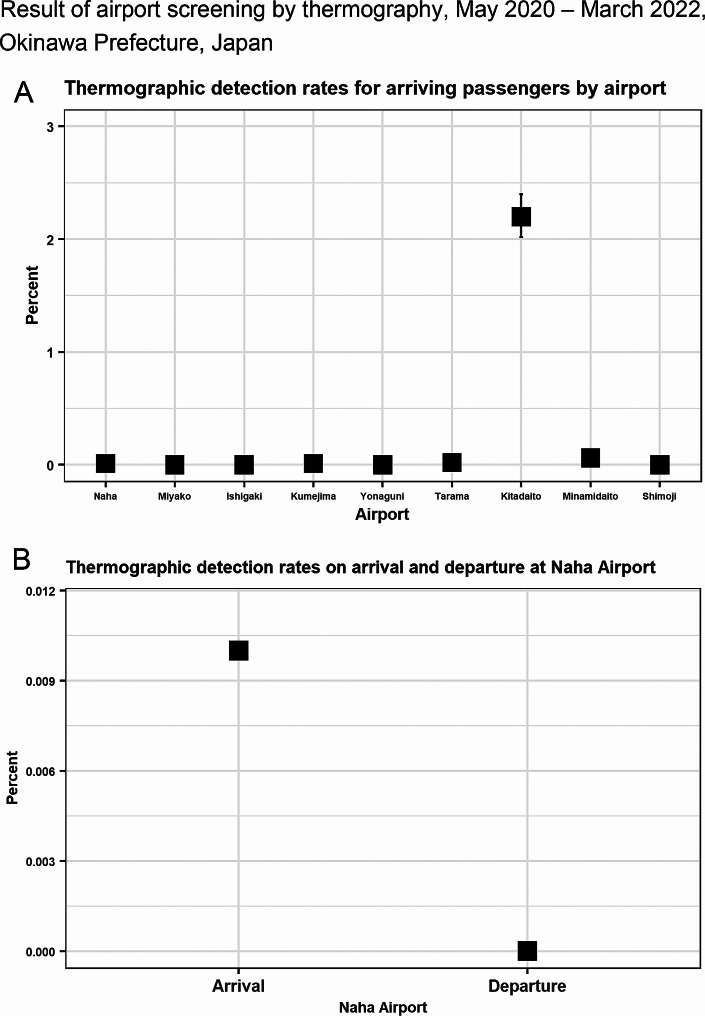
Result of airport screening by thermography, may 2020 – march 2022, Okinawa prefecture, Japan (A) Thermographic detection rates for arriving passengers by airport (B) Thermographic detection rates on arrival and departure at Naha Airport

The results of airport screening from May 2020 to March 2022 are summarized in Fig. 3. The thermographic detection rate for arriving passengers by airport is presented in Fig. 3A, and the highest and second-highest thermographic detection rates were 2.20% (95%CI: 2.02–2.40%) at Kitadaito Airport, and 0.06% (95%CI: 0.03–0.08%) at Minamidaito Airport, respectively. The lowest thermographic detection rate was 0.00% (95%CI: 0.00–0.00%) at Ishigaki Airport, next to 0.00% (95%CI: 0.00-0.01%) at Shimoji Airport. Figure 3B shows the thermographic detection rate at Naha Airport: 0.01% (95%CI: 0.01–0.01%) for arriving passengers and 0.00% (95%CI: 0.00–0.00%) for departing passengers.

**Fig. 4:**
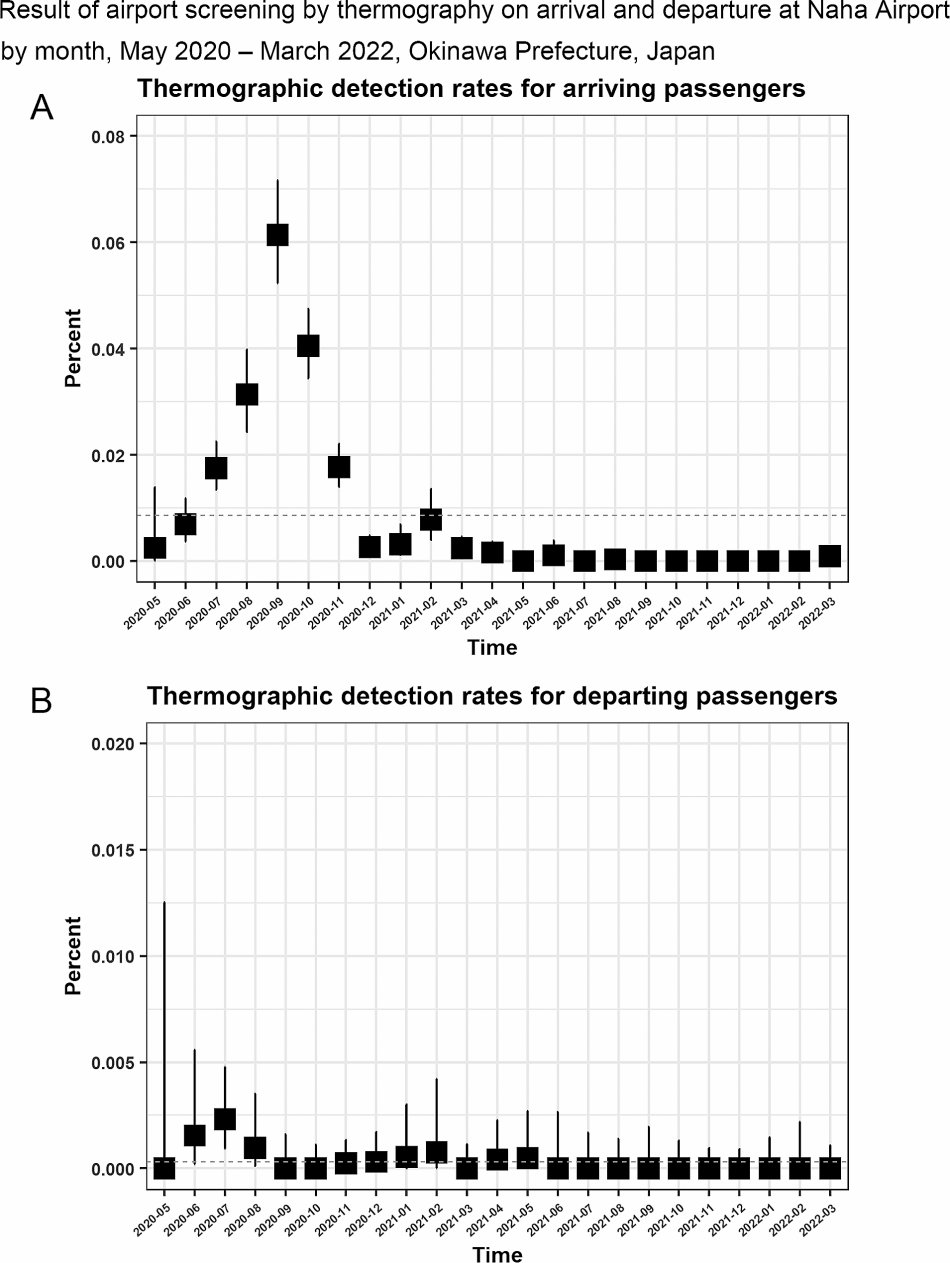
– Result of airport screening by thermography on arrival and departure at Naha Airport stratified by month, may 2020 – march 2022, Okinawa prefecture, Japan (A) Thermographic detection rates for arriving passengers (B) Thermographic detection rates for departing passengers

The monthly thermographic detection rate at Naha Airport for arrivals and departures is shown in Fig. 4. Regarding the thermographic detection rate for arriving passengers, there is a peak from June to November 2020, followed by a smaller peak around February 2021. The highest and second-highest rates were 0.06% (95%CI: 0.05–0.07%) in September 2020, and 0.04% (95%CI: 0.03–0.05%) in October 2020. One peak is seen from June to August 2020 for the thermographic detection rate for departing passengers. Excluding May 2020 due to data scarcity, the top two highest thermographic detection rates were 0.02‰ (95% CI: 0.01–0.05‰) in July 2020 and 0.02‰ (95% CI: 0.00-0.06‰) in June 2020.

Table S1 shows the results of airport screening by month from May 2020 to March 2022, stratified by airport. The total numbers of arriving passengers and those detected by thermography are 9,003,616 and 1162, respectively. The overall thermographic detection rate across the entire duration was 0.01% (95% CI: 0.01–0.01%). The highest and second-highest thermographic detection rates among arriving passengers were 0.06% (95%CI: 0.05–0.07%) in September 2020 and 0.05% (95%CI: 0.04–0.05%) in August 2020.

The thermographic detection rate for arriving passengers at Naha Airport, by setup, for the period from May 2020 to August 14 2020, was 0.01% (95% CI: 0.01–0.01%), 75/698,231, and for the period from August 15 2020 to March 2022 was 0.01% (95 CI: 0.01–0.01%), 509/6,124,498, and these rates were significantly different (proportion test, *p* = 0.04).

Figure S1 displays the number of arriving passengers detected by thermography and the maximum temperature at the observation point nearest the target airport from May 2020 to March 2022 by the airport. There was no apparent correlation between the number of thermographically detected passengers and the maximum temperature, except for Kitadaito airport with a weak correlation coefficient (*r* = 0.3). The results showed that Naha Airport and Kitadaito Airport had an unusually high number of passengers detected by thermography on September 19, 2020 and April 29, 2021, respectively. For Naha Airport, where information is available, the number of passengers determined to have a fever of 37.5 °C or higher was zero on the day in question.

## Discussion

This study examines the effectiveness of airport screening based on thermography alone, conducted during the COVID-19 pandemic in Okinawa Prefecture, where the main entrance to the prefecture is by air. From May 2020 to March 2021, 4.09 million people were reported to pass through airports in Okinawa (Table S1). During the same period, at least 122 people with COVID-19 infection arrived at the airports in Okinawa, but only 10 suspected cases were detected; therefore, the capture rate is estimated to be up to 8.2% (95% CI: 4.00-14.56%). Furthermore, a total of 2,971,198 arriving passengers were screened by thermography at Naha Airport, 572 (0.02%) of whom were identified as potentially having elevated body temperature using thermography, 10 of 568 had a body temperature of 37.5 °C or higher. Considering that airport screening missed over 90% of COVID-19 infected cases, our results indicate that thermography alone may be ineffective for airport screening, suggesting additional measures targeting asymptomatic infections to prevent the influx of infected persons. However, implementing temperature checks at airports may have a certain psychological disincentive for passengers with fever to board, as suggested by the relatively low rate of thermographic detection for departing passengers at airports.

Our result of a fever screening rate of 0.0002% (95%CI: 0.0003–0.0006%) (10 suspected cases /2,971,198 arriving passengers) is consistent with previously reported data of 0.00% (95%CI: 0.00-0.01%) (1 infection /46,016 travelers) in the USA [5]. It is suggested that infectious diseases with long incubation periods reduce the effectiveness of symptom-based screening at airports, especially for domestic flights with only a few hours of flight times. The mean SARS-CoV-2 incubation period between exposure to an infection and the appearance of the first symptoms across variants lies in the range of 4–5 days [24, 25]. Since no law requires RT-PCR testing for febrile passengers detected by fever screening at domestic airports, RT-PCR test results for the 10 febrile patients were not systematically available. In addition, the refusal rate of passengers detected by thermography who did not comply with the temperature measurements using a thermometer was 0.70% (95% CI: 0.19–1.78%). The main reason for refusal is thought to be that if the infection is discovered while traveling with children or on a business trip, the patient is forced to stay at facilities for infected persons for a certain period of time, which is disadvantageous to the patient. The measurement of body temperature, the provision of personal information, and the performance of tests at RT-PCR centers are based on the individual’s consent. Unlike blood sampling, temperature measurement is not invasive and takes only a short time. Although the percentage is low, a certain percentage of febrile passengers among thermographically detected passengers is reported, and it may be worthwhile to consider the possibility of making temperature measurement, RT-PCR testing, and examination of the cause of the fever after thermographic detection mandatory in the future.

When a visitor from outside the prefecture was confirmed to be infected with SARS-CoV-2 while in Okinawa, the visitor had no option but to use medical resources in Okinawa Prefecture. Especially in Okinawa Prefecture, where medical resources are limited. There were reports of a breakdown in the emergency medical system, and testing before boarding is strongly recommended as a border control measure for flights bound for Okinawa Prefecture to prevent an influx of infected passengers, especially when the prevalence of the disease is high [26].

The thermographic detection rate at Kitadaito Airport is significantly higher than at other airports. The number of passengers detected by thermography correlated with max temperature (Figure S1 G), likely influenced by the elevated surface body temperature as the passengers after disembarkation need to walk outdoors to the airport facilities (Figure S2). In addition, there exists a significant difference in thermographic detection rates for arriving passengers at Naha Airport between the setups. Thermography is sensitive to measurement environments, so various factors must be carefully considered when implementing thermography screening. The fever screening projects conducted did not have standards or quality control guidelines to ensure that thermographic equipment and thermometer readings were accurate and consistent, and these need to be developed by the governments.

The thermographic detection rate of departing passengers is significantly lower than that of arriving passengers at Naha Airport. This difference is mainly attributable to the higher thermographic detection rate for arriving passengers from July to November 2020. High outside temperature might have affected the results, but as Figure S1 shows, no correlation is observed between maximum temperature and the frequency of thermographic detection. Since most passengers are tourists, observation of airport screening upon arrival may have increased awareness of precautions, and those with fever may have been screened before the flight at the RT-PCR testing center conducted in the prefecture, but further examination is needed.

Our study is not free from limitations. Firstly, data on screening on ferries and other sea routes are unavailable; therefore, this study does not involve the effect. However, since international cruises, which accounted for most sea passengers, were suspended during COVID-19 [19] and domestic passengers numbered about 10,000 per year [27, 28], the number of infected people entering Okinawa Prefecture by sea is thought to be limited. Besides, there is another inflow route in Okinawa: the U.S. military. According to the latest 2011 report, there appear to be about 4.5 thousand U.S. military personnel and their families in Okinawa Prefecture [29]. While they use the airport on the base for travel to and from the U.S., they use the airports in this study for travel within Japan, and are subject to fever screenings. However, Japan’s Infectious Disease Law is not applicable, and even when infected, the disease is not reported to Japan, thus having little effect on the results of this study.

Secondly, thermography is intended for those with a fever. It has been reported that 17.9% of those infected with SARS-CoV-2 are asymptomatic [30]. Among those with symptoms, it is difficult to capture them before the onset. Additionally, not all those who develop the disease have fever. Even when a fever does develop, antipyretic medications may be taken internally to reduce the fever. These persons cannot be captured by airport screening. However, this study examines the effectiveness of thermography in a real-life context. The conclusion remains the same since these affect the effects toward pushing them down.

Lastly, this is a study of the effectiveness of fever screening at airports. Even with the recommended preboarding RT-PCR testing, reducing the influx of infected persons to zero is impossible. The most reliable way to prevent the influx is to have all arriving passengers stay at designated facilities for a certain period of time, longer than the incubation period. However, it is not feasible for local governments to implement such stringent border control measures, and the socioeconomic impacts on remote islands would be enormous. In addition, whether the influx of infected persons subsequently led to the spread of infection, whether burdened the medical system, and their respective contributions should be verified separately.

Future work will include a detailed cost-effectiveness analysis, a questionnaire survey focusing on natural biological processes such as ovulation and the history of antipyretic medication prior to boarding that affects body temperature, and a study of testing strategies that consider the incubation period, the period between infection and the appearance of fever and other symptoms. The thermography installation and monitoring project at Naha Airport, for which detailed data was obtained, cost approximately 180 million JPY for one year from April 2020 to March 2021. The main breakdown is the cost of commission and purchase of goods [31].

## Conclusion

In conclusion, this study revealed that airport screening for thermography alone missed over 90% of COVID-19 infected cases, strongly indicating that thermography screening is ineffective as a border control measure. Only 10 febrile cases were detected after screening approximately 3 million passengers, which suggests the need to introduce measures targeting asymptomatic infections, especially with long incubation periods. Other countermeasures, e.g. preboarding RT-PCR testing, are highly recommended amid epidemics, satisfying World Health Organization (WHO) Public Health Emergency of International Concern (PHEIC) criteria with pathogen characteristics similar to or exceeding SARS-CoV-2, especially when traveling to rural cities with limited medical resources.

### Electronic supplementary material

Below is the link to the electronic supplementary material.

Supplementary Material 1

Supplementary Material 2

Supplementary Material 3

## Data Availability

The data that support the findings of this study are available from the corresponding author (Dr. Kenji Mizumoto) and the Okinawa Prefecture Government, but restrictions apply to the availability of these data, which were used under license for the current study, and so are not publicly available. Data are however available from the authors upon reasonable request and with the permission of the Okinawa Prefecture Government.

## Abbreviations

COVID-19: Coronavirus disease 2019
RT-PCR: Reverse Transcription Polymerase Chain Reaction
SARS-CoV-2: Severe Acute Respiratory Syndrome Virus 2

## References

[1] Aidan Findlater, Isaac I, Bogoch (2018). Human mobility and the global spread of infectious diseases: a focus on Air Travel. Trends Parasitol.

[2] COVID-19. Current Japanese Border Measures, Ministry of Health, Labour and Welfare, Japan. [cited 2023 July/6] https://www.mhlw.go.jp/stf/covid-19/bordercontrol.html

[3] World Health Organization. Statement on the fifteenth meeting of the IHR. (2005) Emergency Committee on the COVID-19 pandemic. 5 May 2023. [cited 2023 July/6] https://www.who.int/news/item/05-05-2023-statement-on-the-fifteenth-meeting-of-the-international-health-regulations-(2005)-emergency-committee-regarding-the-coronavirus-disease-(covid-19)-pandemic

[4] Normile D (2020). Airport screening is largely futile, research shows. Science.

[5] Dollard P, Griffin I, Berro A, Cohen NJ, Singler K, Haber Y (2020). Risk Assessment and Management of COVID-19 among travelers arriving at designated U.S. airports, January 17-September 13, 2020. MMWR Morb Mortal Wkly Rep.

[6] Billy J, Quilty S Clifford, CMMID nCoV working group; Stefan Flasche, Rosalind M, Eggo. Euro Surveill. Effectiveness of airport screening at detecting travellers infected with novel coronavirus (2019-nCoV). 2020;25(5):2000080. 10.2807/1560-7917 10.2807/1560-7917.ES.2020.25.5.2000080 PMC7014668 32046816

[7] Nishiura H, Kamiya K (2011). Fever screening during the influenza (H1N1-2009) pandemic at Narita International Airport, Japan. BMC Infect Dis.

[8] Praveena J, Gunaratnam S, Tobin H, Seale A, Marich, Jeremy McAnulty (2014). Airport arrivals screening during pandemic (H1N1) 2009 influenza in New South Wales, Australia. Med J Aust.

[9] Sakaguchi H, Tsunoda M, Wada K, Ohta H, Kawashima M, Yoshino Y, Aizawa Y (2012). Assessment of border control measures and community containment measures used in Japan during the early stages of pandemic (H1N1) 2009. PLoS ONE.

[10] Takayama Y, Mizumoto K, Omori R, Yamamoto T (2021). Implementation of SARS-CoV-2 monitoring and screening test using RT-PCR in Okinawa prefecture, Japan. Jxiv (Preprint).

[11] Airport, List. Japan. Ministory of Land, Infrastructure, Transpport and Tourism. Japan. [cited 2023 July/6] https://www.mlit.go.jp/koku/15_bf_000310.html (In Japanese).

[12] Report AMS. 2019. Ministory of Land, Infrastructure, Transport and Tourism. Japan. [cited 2023 July/6] https://www.mlit.go.jp/koku/15_bf_000185.html (In Japanese).

[13] 2019. (Naha Port Statistics. Japan, Corrected Version. December 2022) [cited 2023 July/6] https://nahaport.jp/userfiles/files/00teiseitoukei2019_1.pdf (In Japanese).

[14] Number of visitors. Ishigaki City, Japan. [cited 2023 July/6] https://www.city.ishigaki.okinawa.jp/soshiki/kanko_bunka/survey_statistic/4166.html (In Japanese).

[15] Promotion Plan to Attract Overseas Visitors by Region, Market C. Fiscal Year 2019, Okinawa Convention & Visitors Bureau (OCVB), Okinawa, Japan. https://www.ocvb.or.jp/pages/data/H31_cruisepromotion.pdf (In Japanese).

[16] Tourism survey. 2018 Edition, Okinawa Prefecture, Japan. [cited 2023 July/6] https://www.pref.okinawa.lg.jp/site/bunka-sports/kankoseisaku/kikaku/report/youran/h30kankoyoran.html (In Japanese).

[17] Mizumoto K, Chowell G (2020). Transmission potential of the novel coronavirus (COVID-19) onboard the diamond Princess cruises Ship, 2020. Infect Dis Model.

[18] Maeda H, Sando E, Toizumi M, Arima Y, Shimada T, Tanaka T (2021). Epidemiology of Coronavirus Disease Outbreak among crewmembers on cruise ship, Nagasaki City, Japan, April 2020. Emerg Infect Dis.

[19] Resume accepting international cruises. Press Release. Ministry of Land, Infrastructure, Transpport and Tourism. Japan. [cited 2023 July/6] https://www.mlit.go.jp/report/press/content/001571751.pdf (In Japanese).

[20] International Cruise Ship Arrives in Okinawa for the First Time in Three Years Ishigaki City. March 8, 2023. Ryukyushimpo, Japan. [cited 2023 July/6] https://ryukyushimpo.jp/news/entry-1674064.html (In Japanese).

[21] Summary of Okinawa Prefecture Visitors Statistics for the calendar year 2019. Okinawa Prefecture, Japan. [cited 2023 July/6] https://www.pref.okinawa.jp/site/bunka-sports/kankoseisaku/kikaku/statistics/tourists/documents/r1_reki_gaikyou.pdf (In Japanese).

[22] Hawaii T, Authority USA. [cited 2023 July/6] https://www.hawaiitourismauthority.org/media/4166/2020-01-29-hawaii-visitor-statistics-released-for-december-2019.pdf (In Japanese).

[23] Japan Meteorological Agency. [cited 2023 July/6] https://www.jma.go.jp/jma/index.html (In Japanese).

[24] Galmiche S, Cortier T, Charmet T, Schaeffer L, Chény O (2023). Platenet Cv, al. SARS-CoV-2 incubation period across variants of concern, individual factors, and circumstances of infection in France: a case series analysis from the ComCor study. Lancet Microbe.

[25] Zeng K, Santhya S, Soong A, Malhotra N, Pushparajah D, Thoonet KC (2023). Serial intervals and incubation periods of SARS-CoV-2 Omicron and Delta Variants, Singapore. Emerg Infect Dis.

[26] Okinawa Prefecture Declaration of Medical Emergency (Period: July 21. 2022 - September 29, 2022), September 29, 2022. [cited 2023 July/6] https://www.pref.okinawa.jp/site/chijiko/koho/corona/220721-emergency.html (In Japanese).

[27] Tourism survey. 2020 Edition, Okinawa Prefecture, Japan. [cited 2023 July/6] https://www.pref.okinawa.jp/site/bunka-sports/kankoseisaku/kikaku/report/youran/r1kankoyoran.html (In Japanese).

[28] Tourism survey. 2021 Edition, Okinawa Prefecture, Japan. [cited 2023 July/6] https://www.pref.okinawa.jp/site/bunka-sports/kankoseisaku/kikaku/report/youran/r2kankoyoran.html (In Japanese).

[29] Military US. and Self-Defense Forces Bases in Okinawa (Statistical Data Book), Okinawa Prefecture. July 2022. [cited 2023 July/6] https://www.pref.okinawa.lg.jp/site/chijiko/kichitai/documents/kitinojyoukyou.pdf (The number of military personnel, civilians, and their families is based on interviews with U.S. forces on Okinawa (not provided from 2010 and 2012 to 2021).) (In Japanese).

[30] Mizumoto K, Kagaya K, Zarebski A. Chowell G: Estimating the asymptomatic proportion of coronavirus disease 2019 (COVID-19) cases on board the Diamond Princess cruise ship, Yokohama, Japan, 2020. Euro Surveill. 2020;25(10). 10.2807/1560-7917.ES.2020.25.10.2000180 PMC7078829 32183930

[31] Naha Airport. Thermography installation and monitoring project. Okinawa Prefecture, Japan. [cited 2023 December/23]. https://www.pref.okinawa.lg.jp/site/kikaku/chosei/documents/kikakubu.xlsx (In Japanese)

