# Correction: Assessment of fever screening at airports in detecting domestic passengers infected with SARS-CoV-2, 2020–2022, Okinawa prefecture, Japan

**DOI:** 10.1186/s12879-024-09533-4

**Published:** 2024-06-23

**Authors:** Yoshihiro Takayama, Yining S. Xu, Yusuke Shimakawa, Gerardo Chowell, Masahiro Kozuka, Ryosuke Omori, Ryota Matsuyama, Taro Yamamoto, Kenji Mizumoto

**Affiliations:** 1 Nagasaki University Graduate School of Biomedical Sciences, 1-12-4 Sakamoto, Nagasaki, 852-8523 Japan; 2 Okinawa Prefecture Commission for Epidemiological and Statistical Analysis, Naha-shi, Okinawa Japan; 3 Okinawa Chubu Hospital, 281, Miyazato, Uruma, Okinawa 904-2293 Japan; 4 Department of International Health and Medical Anthropology, Institute of Tropical Medicine, Nagasaki University, 1-12-4 Sakamoto, Nagasaki, 852-8523 Japan; 5 Graduate School of Advanced Integrated Studies in Human Survivability, Kyoto University, 1, Yoshida-Nakaadachi-Cho, Sakyo-Ku, Kyoto, 606-8306 Japan; 6 Unité d’Épidémiologie Des Maladies Émergentes, Institut Pasteur, Université Paris Cité, 25-28 Rue du Docteur Roux, Paris, 75724 France; 7 International Research Center for Medical Sciences, Kumamoto University, 2-2-1, Honjo, Chuo-Ku, Kumamoto, 860-0811 Japan; 8 School of Public Health, Georgia State University, 33 Gilmer Street SE, Atlanta, GA 30303 USA; 9 Division of Bioinformatics, International Institute for Zoonosis Control, Hokkaido University, North 20, West 10 Kita-Ku, Sapporo, Hokkaido 001-0020 Japan; 10 Rakuno Gakuen University, 582, Bunkyodai Midorimachi, Ebetsu, Hokkaido 069-0836 Japan; 11 Hakubi Center for Advanced Research, Kyoto University, Yoshidahonmachi, Sakyo-Ku, Kyoto, 606-8501 Japan; 12 Pasteur International Unit at Kumamoto University / National Center for Global Health and Medicine, Tokyo, Japan

**Correction** **:** **BMC Infect Dis 24, 542 (2024)**

**https://doi.org/10.1186/s12879-024-09427-5**

Following publication of the original article [1], we have been notified that the first affiliation of the first and the eighth author is Nagasaki University Graduate School of Biomedical Sciences, 1-12-4 Sakamoto, Nagasaki, 852-8523, Japan

## References

[1] Takayama (2024). Assessment of fever screening at airports in detecting domestic passengers infected with SARS-CoV-2, 2020–2022, Okinawa prefecture, Japan. BMC Infect Dis.

