# Supplementary figures and images for: Assessment of fever screening at airports in detecting domestic passengers infected with SARS-CoV-2, 2020–2022, Okinawa prefecture, Japan
Source: BMC Infect Dis. 2024 May 30;24:542. doi: 10.1186/s12879-024-09427-5 (PMC11138063; doi:10.1186/s12879-024-09427-5)

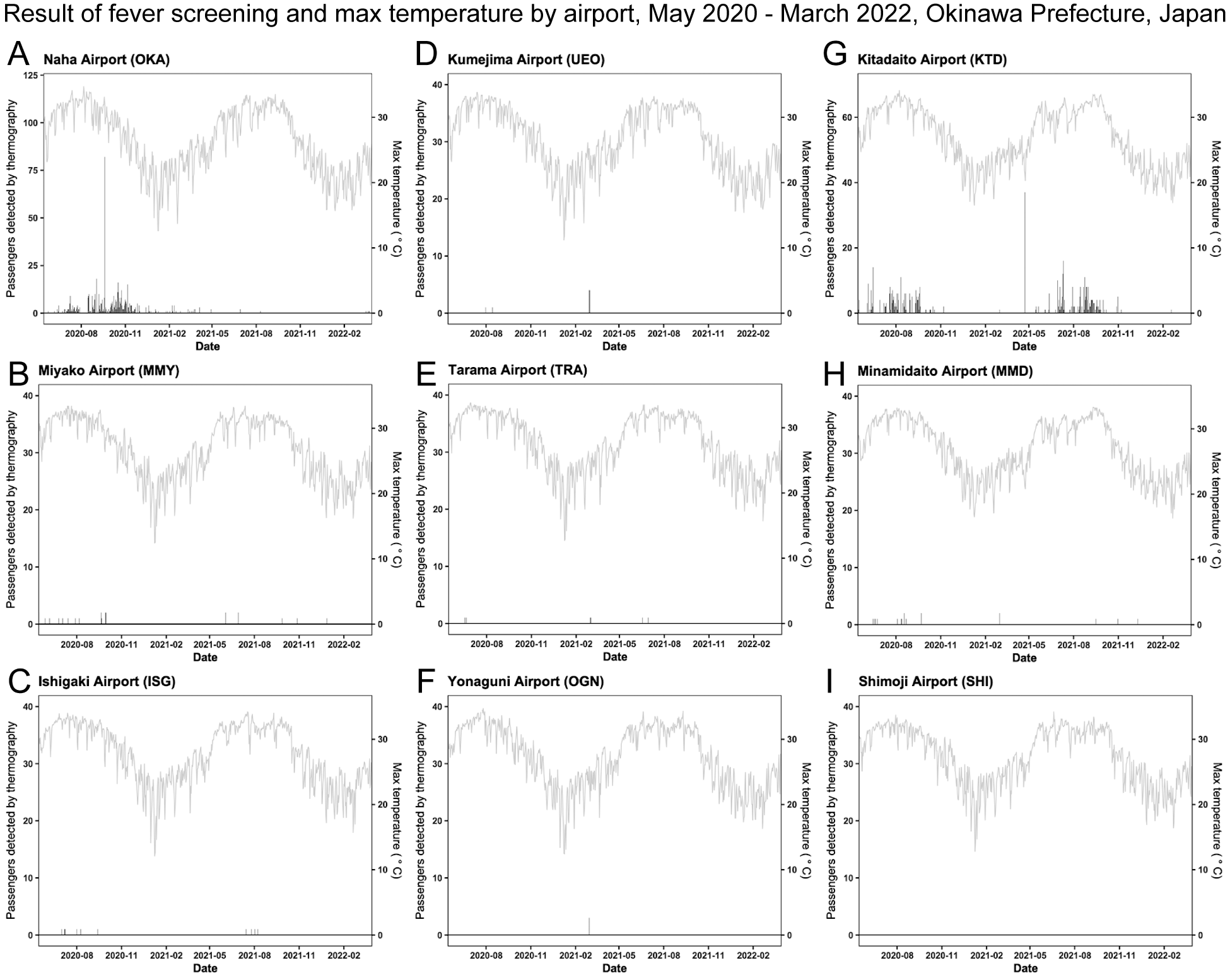

Supplement: Supplementary file 1 — Supplementary Material 1 [file 12879_2024_9427_MOESM1_ESM.png]

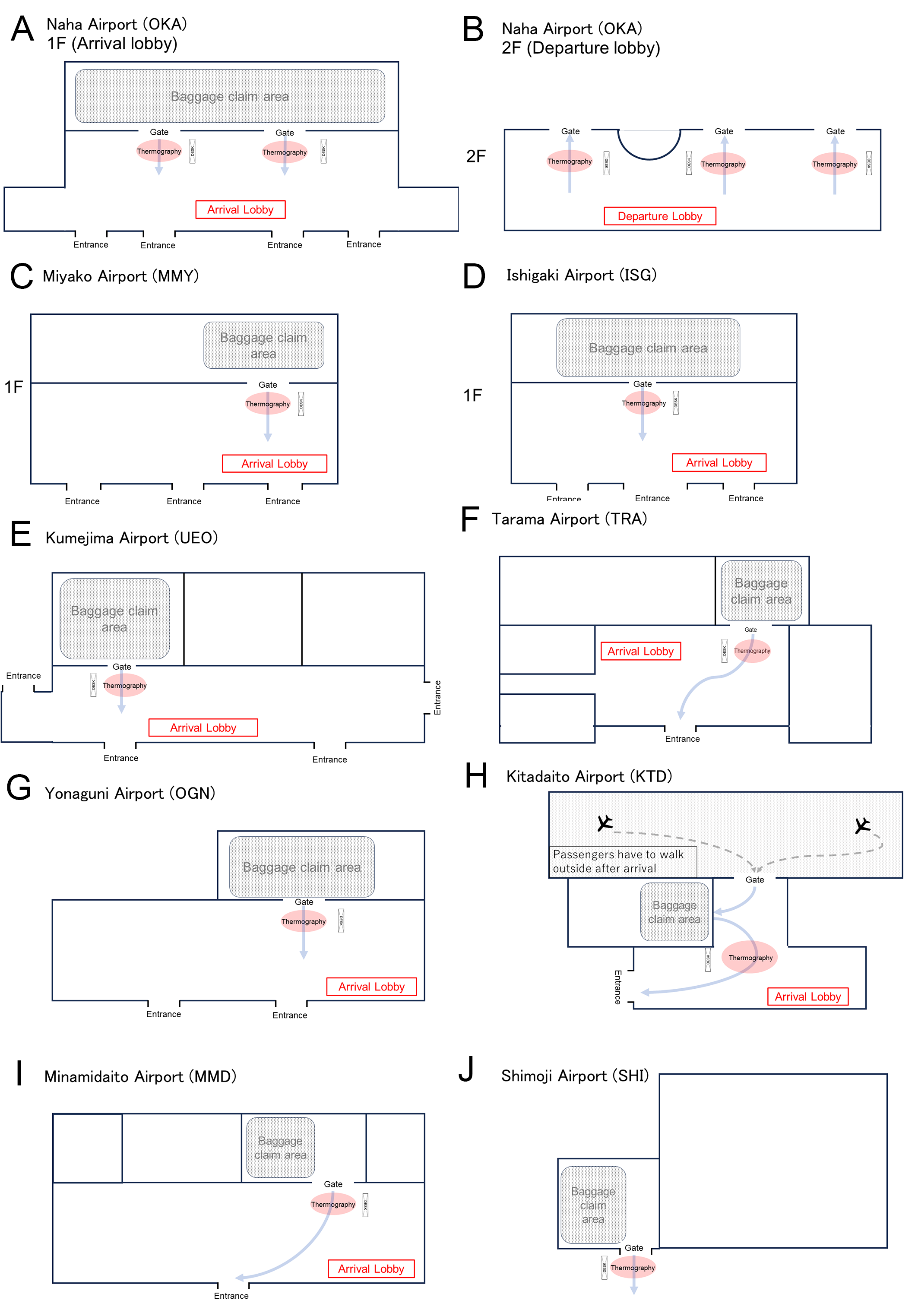

Supplement: Supplementary file 2 — Supplementary Material 2 [file 12879_2024_9427_MOESM2_ESM.png]
