# Supplementary material for: Assessment of fever screening at airports in detecting domestic passengers infected with SARS-CoV-2, 2020–2022, Okinawa prefecture, Japan
Source: BMC Infect Dis. 2024 May 30;24:542. doi: 10.1186/s12879-024-09427-5 (PMC11138063; doi:10.1186/s12879-024-09427-5)
Supplement: Supplementary file 3 — Supplementary Material 3 [file 12879_2024_9427_MOESM3_ESM.docx]

**Supplementary information**

Assessment of fever screening at airports in detecting domestic passengers infected with SARS-CoV-2, 2020-2022, Okinawa Prefecture, Japan

**Supplementary information:**

Below the table titles and figures is the electronic supplementary material.

**Supplementary Tables:**

**Supplementary Table S1**: Number of passengers and those suspected of having an elevated temperature using thermography screening, by month and by airport, May 2020 – Mar 2022, Okinawa Prefecture, Japan

**Supplementary Table S2**. List of thermography equipment and presence of reference standards by airport

**Table S1. Number of passengers and those suspected of having an elevated temperature using thermography screening, by month and by airport, May 2020 – Mar 2022, Okinawa Prefecture, Japan**

| Arriving passengers |  |  |  |  |  |  |  |  |  |  |  |  |  |  |  |  |  |  |  |  |  |  |  |  |  |
| --- | --- | --- | --- | --- | --- | --- | --- | --- | --- | --- | --- | --- | --- | --- | --- | --- | --- | --- | --- | --- | --- | --- | --- | --- | --- |
| Airport |  | 2020 |  |  |  |  |  |  |  | 2021 |  |  |  |  |  |  |  |  |  |  |  | 2022 |  |  | Entire period |
|  |  | May | Jun | Jul | Aug | Sep | Oct | Nov | Dec | Jan | Feb | Mar | Apr | May | Jun | Jul | Aug | Sep | Oct | Nov | Dec | Jan | Feb | Mar |  |
| Naha | Detected^¶^ | 1 | 13 | 61 | 67 | 160 | 155 | 78 | 10 | 6 | 12 | 9 | 5 | 0 | 2 | 0 | 1 | 0 | 0 | 0 | 0 | 0 | 0 | 4 | 584 |
| (OKA)^§^ | Passengers | 40272 | 188016 | 348500 | 213814 | 260670 | 382688 | 441123 | 381648 | 189103 | 154199 | 371165 | 316301 | 237313 | 185618 | 280216 | 305174 | 228561 | 357280 | 471462 | 543996 | 266113 | 229042 | 430455 | 6822729 |
|  | Percent | 0.002 | 0.007 | 0.018 | 0.031 | 0.061 | 0.041 | 0.018 | 0.003 | 0.003 | 0.008 | 0.002 | 0.002 | 0.000 | 0.001 | 0.000 | 0.000 | 0.000 | 0.000 | 0.000 | 0.000 | 0.000 | 0.000 | 0.001 | 0.009 |
| Miyako | Detected | 3 | 2 | 3 | 1 | 7 | 0 | 0 | 0 | 0 | 0 | 0 | 0 | 0 | 2 | 0 | 0 | 1 | 1 | 0 | 1 | 0 | 0 | 0 | 21 |
| (MMY) ^§^ | Passengers | 5398 | 27411 | 47833 | 29817 | 38816 | 55504 | 57034 | 49306 | 22143 | 14764 | 36926 | 36630 | 30543 | 25964 | 22933 | 33699 | 29173 | 47208 | 46509 | 50483 | 27928 | 25964 | 47517 | 809503 |
|  | Percent | 0.056 | 0.007 | 0.006 | 0.003 | 0.018 | 0.000 | 0.000 | 0.000 | 0.000 | 0.000 | 0.000 | 0.000 | 0.000 | 0.008 | 0.000 | 0.000 | 0.003 | 0.002 | 0.000 | 0.002 | 0.000 | 0.000 | 0.000 | 0.003 |
| Ishigaki | Detected | 1 | 0 | 3 | 2 | 1 | 0 | 0 | 0 | 0 | 0 | 0 | 0 | 0 | 0 | 2 | 2 | 0 | 0 | 0 | 0 | 0 | 0 | 0 | 11 |
| (ISG) ^§^ | Passengers | 4962 | 30788 | 61425 | 47601 | 54364 | 84252 | 87856 | 73827 | 28709 | 23942 | 65036 | 60686 | 47404 | 33182 | 56705 | 58720 | 43612 | 69560 | 54901 | 0 | 0 | 36603 | 0 | 1024135 |
|  | Percent | 0.020 | 0.000 | 0.005 | 0.004 | 0.002 | 0.000 | 0.000 | 0.000 | 0.000 | 0.000 | 0.000 | 0.000 | 0.000 | 0.000 | 0.004 | 0.003 | 0.000 | 0.000 | 0.000 | NA | NA | 0.000 | NA | 0.001 |
| Kumejima | Detected | 0 | 0 | 1 | 1 | 0 | 0 | 0 | 0 | 0 | 0 | 8 | 0 | 0 | 0 | 0 | 0 | 0 | 0 | 0 | 0 | 0 | 0 | 0 | 10 |
| (UEO) ^§^ | Passengers | 1212 | 5083 | 7673 | 3746 | 5865 | 8220 | 8871 | 7983 | 4768 | 4223 | 6706 | 5383 | 5293 | 3967 | 5313 | 0 | 4681 | 6975 | 7747 | 8640 | 4662 | 4614 | 6220 | 127845 |
|  | Percent | 0.000 | 0.000 | 0.013 | 0.027 | 0.000 | 0.000 | 0.000 | 0.000 | 0.000 | 0.000 | 0.119 | 0.000 | 0.000 | 0.000 | 0.000 | #DIV/0 | 0.000 | 0.000 | 0.000 | 0.000 | 0.000 | 0.000 | 0.000 | 0.008 |
| Tarama | Detected | 0 | 2 | 0 | 0 | 0 | 0 | 0 | 0 | 0 | 0 | 2 | 0 | 0 | 1 | 0 | 0 | 0 | 0 | 0 | 0 | 0 | 0 | 0 | 5 |
| (TRA) ^§^ | Passengers | 319 | 1053 | 1460 | 498 | 962 | 1364 | 1495 | 1579 | 1062 | 659 | 1403 | 1156 | 870 | 864 | 977 | 1077 | 636 | 1378 | 1685 | 1746 | 1034 | 1160 | 1378 | 25815 |
|  | Percent | 0.000 | 0.190 | 0.000 | 0.000 | 0.000 | 0.000 | 0.000 | 0.000 | 0.000 | 0.000 | 0.143 | 0.000 | 0.000 | 0.116 | 0.000 | 0.000 | 0.000 | 0.000 | 0.000 | 0.000 | 0.000 | 0.000 | 0.000 | 0.019 |
| Yonaguni | Detected | 0 | 0 | 0 | 0 | 0 | 0 | 0 | 0 | 0 | 0 | 3 | 0 | 0 | 0 | 0 | 0 | 0 | 0 | 0 | 0 | 0 | 0 | 0 | 3 |
| (OGN) ^§^ | Passengers | 481 | 1962 | 3136 | 2198 | 2746 | 3348 | 4146 | 3306 | 2517 | 2372 | 3668 | 2745 | 2761 | 1824 | 2412 | 2105 | 1858 | 2731 | 3303 | 4067 | 2946 | 2622 | 2554 | 61808 |
|  | Percent | 0.000 | 0.000 | 0.000 | 0.000 | 0.000 | 0.000 | 0.000 | 0.000 | 0.000 | 0.000 | 0.082 | 0.000 | 0.000 | 0.000 | 0.000 | 0.000 | 0.000 | 0.000 | 0.000 | 0.000 | 0.000 | 0.000 | 0.000 | 0.005 |
| Kitadaito | Detected | 5 | 45 | 60 | 63 | 50 | 7 | 2 | 0 | 0 | 0 | 1 | 37 | 5 | 10 | 73 | 76 | 58 | 8 | 6 | 0 | 0 | 1 | 0 | 507 |
| (KTD) ^§^ | Passengers | 320 | 1117 | 1109 | 630 | 1006 | 1090 | 1191 | 1049 | 1065 | 770 | 1180 | 928 | 1089 | 709 | 822 | 909 | 974 | 1279 | 1229 | 1272 | 1057 | 1049 | 1152 | 22996 |
|  | Percent | 1.563 | 4.029 | 5.410 | 10.000 | 4.970 | 0.642 | 0.168 | 0.000 | 0.000 | 0.000 | 0.085 | 3.987 | 0.459 | 1.410 | 8.881 | 8.361 | 5.955 | 0.625 | 0.488 | 0.000 | 0.000 | 0.095 | 0.000 | 2.205 |
| Minamidaito | Detected | 4 | 4 | 0 | 6 | 2 | 0 | 0 | 0 | 0 | 0 | 2 | 0 | 0 | 0 | 0 | 0 | 1 | 0 | 1 | 1 | 0 | 0 | 0 | 21 |
| (MMD) ^§^ | Passengers | 321 | 1577 | 1859 | 1130 | 1802 | 1974 | 1989 | 1969 | 1825 | 1388 | 2121 | 1497 | 1737 | 1181 | 1418 | 1388 | 1294 | 1925 | 2163 | 2139 | 1630 | 1562 | 1932 | 37821 |
|  | Percent | 1.246 | 0.254 | 0.000 | 0.531 | 0.111 | 0.000 | 0.000 | 0.000 | 0.000 | 0.000 | 0.094 | 0.000 | 0.000 | 0.000 | 0.000 | 0.000 | 0.077 | 0.000 | 0.046 | 0.047 | 0.000 | 0.000 | 0.000 | 0.056 |
| Shimoji | Detected | 0 | 0 | 0 | 0 | 0 | 0 | 0 | 0 | 0 | 0 | 0 | 0 | 0 | 0 | 0 | 0 | 0 | 0 | 0 | 0 | 0 | 0 | 0 | 0 |
| (SHI) ^§^ | Passengers | 0 | 832 | 5788 | 4930 | 6354 | 8404 | 10027 | 0 | 0 | 0 | 0 | 0 | 0 | 6814 | 0 | 0 | 0 | 13151 | 0 | 11480 | 3184 | 0 | 0 | 70964 |
|  | Percent | NA | 0.000 | 0.000 | 0.000 | 0.000 | 0.000 | 0.000 | NA | NA | NA | NA | NA | NA | 0.000 | NA | NA | NA | 0.000 | NA | 0.000 | 0.000 | NA | NA | 0.000 |
| All airports | Detected | 14 | 66 | 128 | 140 | 220 | 162 | 80 | 10 | 6 | 12 | 25 | 42 | 5 | 15 | 75 | 79 | 60 | 9 | 7 | 2 | 0 | 1 | 4 | 1162 |
|  | Passengers | 53285 | 257839 | 478783 | 304364 | 372585 | 546844 | 613732 | 520667 | 251192 | 202317 | 488205 | 425326 | 327010 | 260123 | 370796 | 403072 | 310789 | 501487 | 588999 | 623823 | 308554 | 302616 | 491208 | 9003616 |
|  | Percent | 0.026 | 0.026 | 0.027 | 0.046 | 0.059 | 0.030 | 0.013 | 0.002 | 0.002 | 0.006 | 0.005 | 0.010 | 0.002 | 0.006 | 0.020 | 0.020 | 0.019 | 0.002 | 0.001 | 0.000 | 0.000 | 0.000 | 0.001 | 0.013 |
| Departing passengers |  |  |  |  |  |  |  |  |  |  |  |  |  |  |  |  |  |  |  |  |  |  |  |  |  |
|  |  | 2020 |  |  |  |  |  |  |  | 2021 |  |  |  |  |  |  |  |  |  |  |  | 2022 |  |  | Entire period |
|  |  | May | Jun | Jul | Aug | Sep | Oct | Nov | Dec | Jan | Feb | Mar | Apr | May | Jun | Jul | Aug | Sep | Oct | Nov | Dec | Jan | Feb | Mar |  |
| Naha | Detected | 0 | 2 | 7 | 2 | 0 | 0 | 1 | 1 | 1 | 1 | 0 | 1 | 1 | 0 | 0 | 0 | 0 | 0 | 0 | 0 | 0 | 0 | 0 | 17 |
| (OKA) ^§^ | Passengers | 29454 | 129412 | 302382 | 205400 | 229587 | 331297 | 416706 | 326659 | 185348 | 132524 | 324634 | 244340 | 207164 | 139745 | 220318 | 264525 | 188425 | 281253 | 389559 | 408337 | 251066 | 169017 | 335831 | 5712983 |
|  | Percent | 0.0000 | 0.0015 | 0.0023 | 0.0010 | 0.0000 | 0.0000 | 0.0002 | 0.0003 | 0.0005 | 0.0008 | 0.0000 | 0.0004 | 0.0005 | 0.0000 | 0.0000 | 0.0000 | 0.0000 | 0.0000 | 0.0000 | 0.0000 | 0.0000 | 0.0000 | 0.0000 | 0.0003 |

^§^ International Airport Transport Association code

^¶^ Number of passengers who were detected by thermography

**Table S2. List of thermography equipment and presence of reference standards by airport**

|  | Naha Airport (OKA) | Other airports |
| --- | --- | --- |
| Detailed information on RT-PCR | Not available | Not available |
| Thermography Equipment  Type (Product No.) | For arriving passengers   - Dome-type AI Thermal Camera (DS-2TD1217B-3/PA)   For departing passengers   - Dome-type AI Thermal Camera (DS-2TD1217B-3/PA) - Dome-type AI Thermal Camera (IR-DC-TH20) | For arriving passengers   - Dome-type AI Thermal Camera (DS-2TD1217B-6/PA) |
| Thermometer | Non-contact thermometer directed to the forehead or wrists. | |
| Presence of reference standards/standard measurement protocols | No | |
| Quality control | The contractor was implementing quality control on its own.  Employees at Naha and other airports confirmed that there was no difference between their body temperatures measured by thermography and those measured by non-contact thermometers as a standard. | |

# Supplementary Figures:

**Figure S1. Results of fever screening and max temperature by the airport, May 2020 – March 2022, Okinawa Prefecture, Japan.**





(A) Naha Airport (OKA), (B) Miyako Airport (MMY), (C) Ishigaki Airport (ISG), (D) Kumejima Airport (UEO), (E) Tarama Airport (TRA), (F) Yonaguni Airport (OGN), (G) Kitadaito Airport (KTD), (H) Minamidaito (MMD), (I) Shimoji Airport (SHI)

**Figure S2. Simplified map of the airports and thermography locations by airport, Okinawa Prefecture**


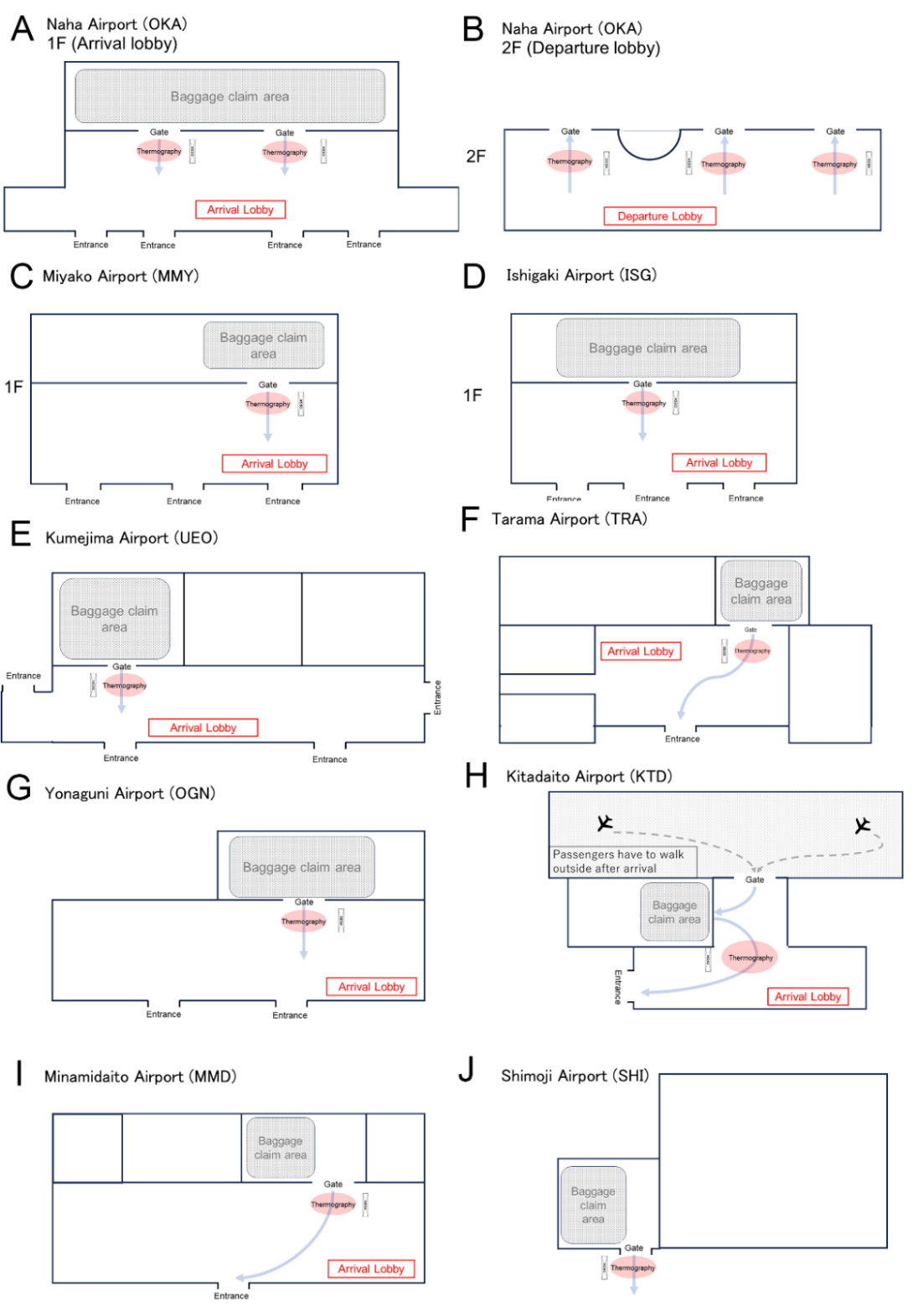


(A) Arrival lobby at Naha Airport (OKA), (B) Departure lobby at Naha Airport (OKA)

(C) Miyako Airport (MMY), (D) Ishigaki Airport (ISG), (E) Kumejima Airport (UEO), (F) Tarama Airport (TRA), G) Yonaguni Airport (OGN), (H) Kitadaito Airport (KTD), (I) Minamidaito (MMD), (J) Shimoji Airport (SHI)

Arrow indicate the flow of passengers movement. The flow is shown for arriving passengers from the baggage claim to the exit. The locations measured by the thermoscanner are indicated by the red-colored areas.
